# Association of multiple blood metals with thyroid function in general adults: A cross−sectional study

**DOI:** 10.3389/fendo.2023.1134208

**Published:** 2023-03-27

**Authors:** Yanshi Ye, Ye Li, Qintao Ma, Ying Li, Huixian Zeng, Yaosheng Luo, Yongqian Liang, Lan Liu, Lingling Liu, Xu Lin, Genfeng Yu, Cheng Song, Heng Wan, Jie Shen

**Affiliations:** Department of Endocrinology and Metabolism, Shunde Hospital, Southern Medical University (The First People’s Hospital of Shunde), Foshan, Guangdong, China

**Keywords:** thyroid hormones, thyroid stimulating hormone, FT3:FT4 ratio, thyrotropin T4 resistance index, zinc, iron, copper, manganese

## Abstract

**Introduction:**

Thyroid function has a large impact on humans’ metabolism and is affected by iodine levels, but there is a scarcity of studies that elucidate the association between thyroid function and other elements.

**Methods:**

We performed a cross-sectional study on 1,067 adults to evaluate the associations of the common essential metals with thyroid function in adults living in an iodine-adequate area of China. Serum free thyroxine (FT4), free triiodothyronine (FT3), thyroid stimulating hormone (TSH), and blood metals (zinc, iron, copper, magnesium, manganese, and calcium) were measured. Further, the thyroid hormone sensitivity indexes, FT3:FT4 ratio, and thyrotropin T4 resistance index (TT4RI) were calculated. Linear regression, quantile g-computation, and Bayesian kernel machine regression methods were used to explore the association of metals with thyroid function.

**Results:**

We found that the TSH levels correlated with copper (negative) and zinc (positive). Iron and copper were positively associated with FT3 and FT4 levels, respectively. Iron (positive) and copper (negative) were correlated with the FT3:FT4 ratio. Furthermore, we found that manganese was inversely correlated with TT4RI, while zinc was positively correlated.

**Discussion:**

Our findings suggest that manganese, iron, copper, and zinc levels were strongly correlated with thyroid function, and patients with thyroid disorders are recommended to measure those metals levels.

## 1 Introduction

Thyroid function is crucial in fetal development, adolescent growth, and metabolic homeostasis. In adults, the thyroid hormone mainly affects metabolism through actions in the brain, skeletal muscle, liver, pancreas, and adipose tissue (1). Thyroid function disorder is associated with obesity, diabetes, metabolic syndrome, and cardiovascular diseases (2, 3). A recent epidemiological investigation in China reported that although the prevalence of thyroid disorders has decreased by implementing mandatory universal salt iodization, the total prevalence of thyroid disorders was 15.22% in 2016 (4). Therefore, it is necessary to investigate which environmental factors other than iodine deficiency affect thyroid function.

Generally, serum concentrations of thyroid-stimulating hormone (TSH), thyroxine (T4), and triiodothyronine (T3) are used to assess thyroid function. Physiologically, T4 and small amounts of T3 are synthesized and released by thyroid follicular cells that are controlled by TSH, which is produced in the anterior pituitary gland and regulated by thyrotropin-releasing hormone (TRH). Approximately 80% of T3 is transformed from T4 through deiodination in peripheral tissue. In turn, the circulating thyroid hormone inhibits the production of TSH and TRH, which is called a negative feedback loop and is critical for maintaining a proper serum concentration of the thyroid hormone (5). The thyrotropin T4 resistance index (TT4RI) was proposed to estimate the central sensitivity of the thyroid hormone. A high TT4RI value was found to be related to an increased risk of hyperuricemia, diabetes, and metabolic syndrome (6, 7). In addition, both blood T3 and T4 have a high affinity with the plasma-binding proteins, and only free T3 (FT3) and free T4 (FT4) are biologically active and available for effector cells (8). The FT3:FT4 ratio is regarded as an index of types 1 and 2 deiodinase (D1 and D2) activity, and it is suggested that a low FT3:FT4 ratio has a high risk of mortality (9, 10).

Essential metals, such as zinc (Zn), iron (Fe), magnesium (Mg), calcium (Ca), manganese (Mn), selenium (Se), and copper (Cu), are essential for various normal physiological processes and functions. It is well established that alteration of essential metal levels may affect the risk of thyroid diseases, including hypothyroidism, hyperthyroidism, and thyroid cancer (11–13). A case–control study revealed that low blood concentrations of Ca, Mg, Zn, and Se combined with reduced iodine concentration in urine are a high risk for goiter (14). Clinical trials have concluded that supplementing Fe and iodized salt simultaneously could decrease the prevalence of hypothyroidism and goiter in goitrous children with iron deficiency compared with only supplementing iodized salt (15, 16). Serum Cu levels have also been reported to be associated with hyperthyroidism and thyroid autoimmunity, and a low serum Cu level is related to a higher incidence of ophthalmopathy in patients with Graves’ disease (17). However, essential metals are potentially toxic at high concentrations. For example, a cross-sectional study found that Mn was inversely related to the levels of thyroid hormone concentration among pregnant Chinese women (18). In the general U.S. population, the level of Cu is related to increased levels of thyroid hormones in both men and women, while Zn is associated with decreased thyroid hormones in men (19). In the current literature, environmental factors affecting thyroid function have been studied more in thyroid disease patients, pregnant women, and children, and fewer investigations have been conducted on the general population.

This cross-sectional study aimed to evaluate the associations of the common essential metals (Zn, Cu, Fe, Mg, Ca, and Mn) with thyroid function in adults living in an iodine-adequate area of China. Except for the TSH, FT3, and FT4 levels, we adopted the FT3:FT4 ratio and TT4RI to indicate the peripheral and central sensitivities of the thyroid hormone. Since individuals are exposed to multiple metals simultaneously, the overall effect of the metal mixture on human health needs to be assessed (20, 21). Thus, besides linear regression, we also applied quantile g-computation (QGC) and Bayesian kernel machine regression (BKMR) to evaluate the association of individual metals and metal mixtures with the above thyroid indicators at the same time. The results of our study may provide some suggestions for supplementing insufficient elements or avoiding exposure to excess elements and preventing thyroid disorders in a general population.

## 2 Methods

### 2.1 Study population

**Figure 1:**
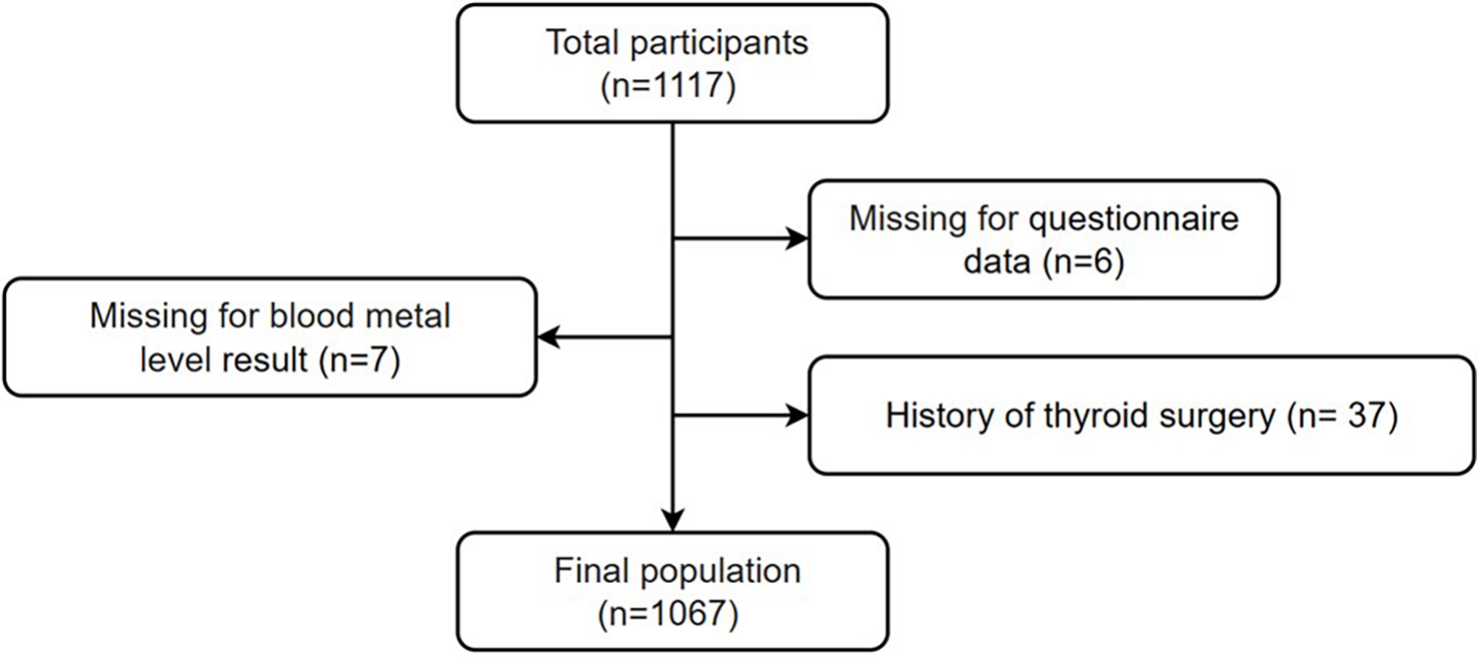
Flowchart of the final population included in this study.

This study analyzed 1,117 adults (aged ≥18 years) who lived in Lecong Town, Shunde District, Guangdong, China (an iodine-adequate area) (4). They were recruited using a stratified cluster sampling method and included in a cross-sectional study about the risks of metabolic diseases (www.chictr.org.cn, ChiCTR2100054130) in 2021. We excluded those with missing questionnaire data (*n* = 6) or blood metal level results (*n* = 7) and those with a history of thyroid surgery (*n* = 37). Ultimately, 1,067 participants were included in the analysis (**Figure 1**). Participants were informed about the purpose and procedure of the investigation, and they provided signed informed consent. The study was approved by the Ethics Committee of Shunde Hospital, Southern Medical University, Shunde, Foshan, China (20211103).

### 2.2 Laboratory assays

Fasting blood was collected from participants between 08:00 and 10:00 after an overnight fast and transported to the central laboratory within 2–4 h. The blood samples were used to detect the concentrations of metals, including Zn, Fe, Ca, Cu, Mg, and Mn, using inductively coupled plasma mass spectrometry (ICAP-RQ, Thermofisher Scientific, USA). The laboratory reference ranges were as follows: Zn (5.0–7.5 mg/L), Fe (421.1–660.8 mg/L), Cu (749.3–1,394.5 μg/L), Mg (26.9–49.4 mg/L), Mn (6.6–21.6 μg/L), and Ca (56.8–76.0 mg/L). Levels of TSH, FT3, and FT4 were assessed using chemiluminescent immunoassays (Siemens, Centaur XPT, Erlangen, Germany), and the laboratory reference ranges were as follows: TSH (0.49–4.91 μIU/ml), FT3 (3.28–6.47 pmol/L), and FT4 (7.64–16.03 pmol/L). For the covariates, the total cholesterol (TC) and triglycerides (TG) were measured using Mindray, BS-800 (Shenzhen, China). Glycated hemoglobin (HbA1c) was detected using high-performance liquid chromatography (TOSOH, HLC-723 G8, Tokyo, JAPAN), and urinary iodine was detected using the stripping voltammetry analysis method (Shenrui SR-L100, Wuxi, China).

### 2.3 Data collection and definitions

The demographic characteristics, lifestyle, and medical history of the patients were obtained using a direct questionnaire. Their body weight, height, and blood pressure were measured according to the standard protocols. Their body mass index (BMI) (kg/m^2^) was calculated using the patient’s weight and height. Hypertension was defined as systolic blood pressure (SBP) ≥140 mmHg or diastolic blood pressure (DBP) ≥90 mmHg (22). Diabetes was defined as HbA1c ≥6.5%. The three smoking statuses were “never smoked,” “used to smoke,” and “currently smokes” (has smoked at least 100 cigarettes and still smokes).

### 2.4 Indexes of thyroid hormones sensitivity

Two indexes were calculated to evaluate the sensitivity of the thyroid hormone according to previous studies (6, 7). The FT3:FT4 ratio reflected the response of peripheral tissue to the thyroid hormone and was calculated as FT3 (pmol/L)/FT4 (pmol/L). The thyrotropin T4 resistance index (TT4RI) is an indicator that reflects the sensitivity of the hypothalamic–pituitary–thyroid axis (HPT axis) to the thyroid hormone and is calculated as TT4RI = FT4 (pmol/L) * TSH (mIU/L). Low values of TT4RI indicate that less TSH was released than expected according to the actual FT4 level. High TT4RI values indicate a higher TSH level than expected, according to the actual FT4 level.

### 2.5 Statistical analysis

Statistical analyses were performed using the software IBM SPSS Statistics, version 26.0 (IBM, USA) or R version 4.2.1. A *p*-value (two-sided) of <0.05 was statistically significant. Continuous variables were expressed as the mean ± standard deviation (SD) or the median with an interquartile range (25%, 75%), and the categorical variables were presented as percentages (%). Continuous variables with skewed distributions (Ca, Mg, Mn, Fe, Cu, Zn, FT3, FT4, TSH, FT3:FT4 ratio, and TT4RI) were ln transformed for analysis. All the models were adjusted for age, gender, BMI, smoking status, TG, TC, hypertension, diabetes, and urinary iodine.

#### 2.5.1 Linear regression

We used a linear regression to investigate the association between metals and thyroid function biomarkers (FT3, FT4, TSH, FT3:FT4 ratio, and TT4RI). Metal levels were treated as continuous variables and all metals were put into the model together.

#### 2.5.2 Bayesian kernel machine regression model

We applied the BKMR model to further explore the relationship between thyroid function and a single metal or metal mixture. The BKMR model was also used to explore the interactions among metals. We fitted the BKMR model with variable selection and estimated the posterior inclusion probability (PIP) for each of the metals. Higher values of PIP present more importance for outcomes, and a PIP value above 0.5 is usually used to determine the impactive factors (23). The BKMR package of R was used for the analysis.

#### 2.5.3 Quantile g-computation model

We used the QGC model to assess the association between the metal mixture and thyroid function biomarkers and the relative contributions of different metals to these biomarkers. We identified a metal as “influential” if the absolute value of the metal’s weight was greater than 0.333, which was derived by dividing 2 (sum of the absolute weights) by 6 (total number of metals in the mixture) to represent the value of the weight when all metals contributed equally (20). The analysis was carried out with the help of the R package, qgcomp.

## 3 Results

### 3.1 Study population characteristics

The general characteristics of the 1,067 subjects, composed of 465 men and 602 women, are presented in **Table 1**. The average age of the participants was 49.56 years, and 14.3% were current smokers. Most subjects (84.4%) had a BMI range of 18 to 28 kg/m^2^. The prevalence of hypertension and diabetes was 45.6% and 9.5%, respectively. The median urinary iodine concentration was 196 μg/g. The median concentration of TG was 1.23 mmol/L, and the mean concentration of TC was 5.45 mmol/L.

**Table 1:** Characteristics of study population (*n* = 1,067).

Parameter	
*N*	1,067
Age (years)	49.56 ± 14.437
Gender (%)	
Men	465 (43.6%)
Women	602 (56.4)
Smoking status (%)	
Current smokers	153 (14.3%)
Ex-smokers	51 (4.8%)
Non-smokers	863 (80.9%)
BMI (kg/m^2^)	
<18	29 (2.7%)
18–24	552 (51.7%)
24–28	349 (32.7%)
>28	137 (12.8%)
TG (mmol/L)	1.23 (0.88,1.83)
TC (mmol/L)	5.45 ± 1.15
Hypertension (%)	487 (45.6%)
Diabetes (%)	101 (9.5%)
Urinary iodine (μg/g)	196.00 (146.00,262.00)
FT3 (pmol/L)	5.53 ± 0.83
FT4 (pmol/L)	11.37 ± 1.71
TSH (μIU/ml)	1.56 (1.07,2.32)
FT3:FT4 ratio	0.50 ± 0.10
TT4RI	17.80 (12.23,26.02)
Ca (mg/L)	62.97 ± 5.85
Mg (mg/L)	41.78 ± 4.46
Mn (μg/L)	13.14 ± 4.07
Fe (mg/L)	506.11 ± 57.20
Cu (μg/L)	884.86 ± 124.53
Zn (mg/L)	6.33 ± 0.93

Data are expressed as the mean ± SD or median (25th and 75th quartiles) or number (%).

BMI, body mass index; TG, triglycerides; TC, total cholesterol; FT3, free triiodothyronine; FT4, free thyroxine; TSH, thyrotropin; TT4RI, thyrotropin T4 resistance index.

Regarding the thyroid function biomarkers, the mean concentrations of serum FT3 and FT4 were 5.53 pmol/L and 11.37 pmol/L, respectively. The median concentration of TSH was 1.56 μIU/ml. The mean value of the FT3:FT4 ratio was 0.5, and the median value of TT4RI was 17.80.

**Figure 2:**
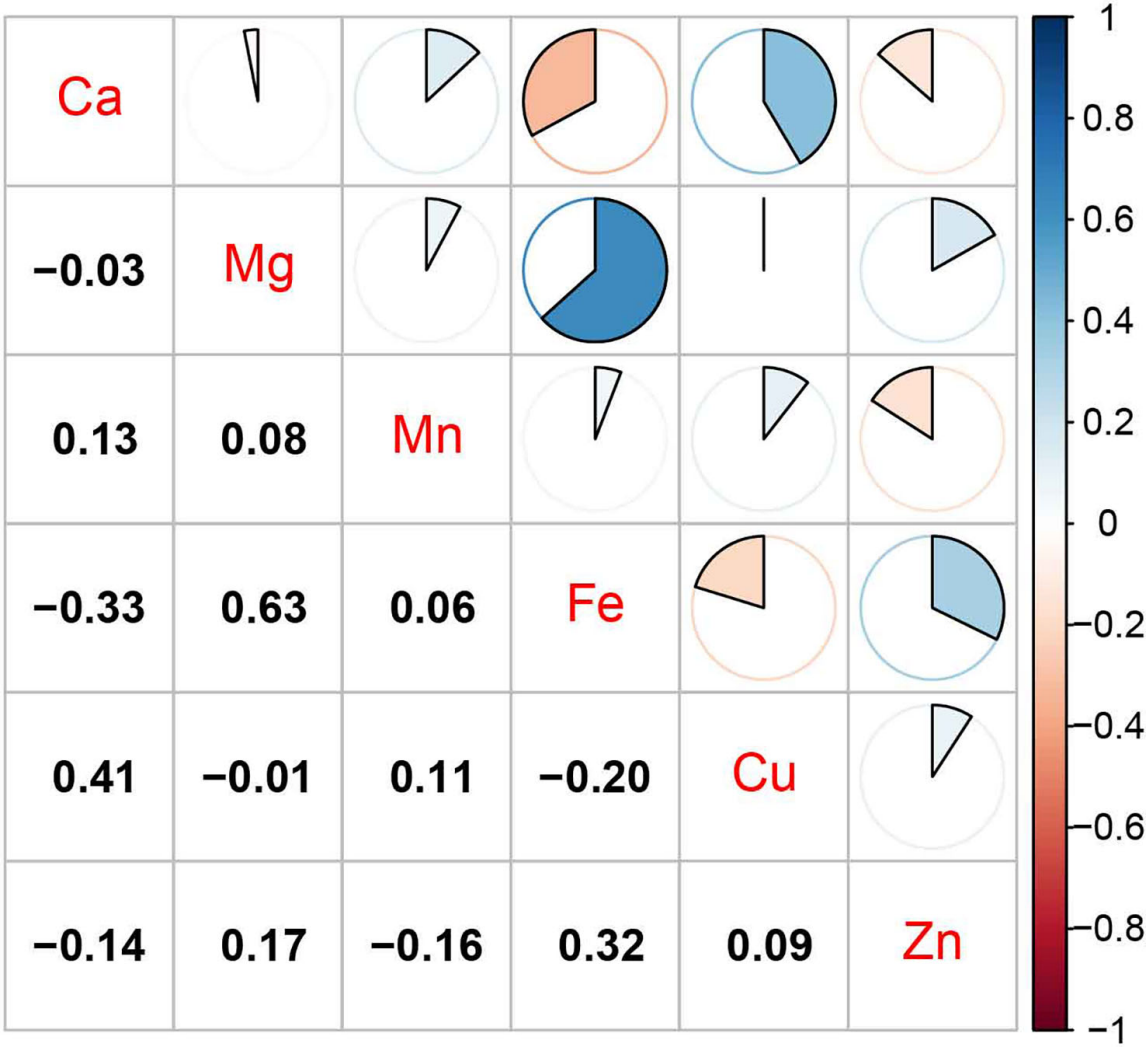
Spearman’s correlations among seven metals’ levels (ln).

The mean concentrations of blood Ca, Mg, Mn, Fe, Cu, and Zn were 62.97 mg/L (SD: 5.85), 41.78 mg/L (SD: 4.46), 13.14 μg/L (SD: 4.07), 506.11 mg/L (SD: 57.2), 884.86 μg/L (SD: 124.53), and 6.33 mg/L (SD: 0.93), respectively. The correlations between the blood concentrations of different metals were not very strong; the strongest correlation was observed between Mg and Fe (*r*s: 0.63), and a moderate correlation was found between Ca and Cu (*r*s: 0.41). The remaining correlations were weak (*r*s: −0.33–0.32) (**Figure 2**).

### 3.2 The association between selected metals and thyroid function biomarkers

Linear regression models were applied to estimate the associations between selected metals and FT3, FT4, TSH, FT3:FT4 ratio, and TT4RI in the combined exposure model (**Table 2**). Blood Fe was positively correlated with FT3 (*β* = 0.169, 95% CI: 0.064, 0.274) and the FT3:FT4 ratio (*β* = 0.153, 95% CI: 0.004, 0.303). Additionally, blood Cu was significantly positively associated with FT3 (*β* = 0.068, 95% CI: 0.000,0.136) and FT4 (*β* = 0.164, 95% CI: 0.087,0.241). Blood Cu is also inversely linked to TSH (*β* = −0.440, 95% CI: −0.847, −0.007) and had a borderline positive correlation with the FT3:FT4 ratio (*β* = −0.096, 95% CI: −0.192, 0.001, *p* = 0.052). Also, there were links between blood Zn and serum FT3 (*β* = −0.065, 95% CI: −0.122, −0.007), as well as serum TSH (*β* = 0.394, 95% CI: 0.026, 0.762). Furthermore, blood Zn had a borderline positive correlation with FT4 (*β* = −0.058, 95% CI: −0.124, 0.007, *p* = 0.082) and TT4RI (*β* = 0.336, 95% CI: −0.015, 0.687, *p* = 0.060). Blood Mn had a borderline negative correlation with TT4RI (*β* = −0.147, 95% CI: −0.312, 0.017, *p* = 0.079).

**Table 2:** Linear regression analysis for the correlation of thyroid function biomarkers and metals.

Metals	lnFT3		lnFT4		lnTSH		lnFT3:FT4 ratio		lnTT4RI	
	*β* (95% CI)	*p*	*β* (95% CI)	*p*	*β* (95% CI)	*p*	*β* (95% CI)	*p*	*β* (95% CI)	*p*
lnCa	−0.068 (−0.171,0.034)	0.190	−0.088 (−0.204,0.029)	0.140	0.124 (−0.529,0.778)	0.709	0.019 (−0.126,0.165)	0.795	0.037 (−0.585,0.659)	0.907
lnMg	−0.030 (−0.131,0.071)	0.555	−0.043 (−0.158,0.071)	0.458	−0.074 (−0.718,0.571)	0.823	0.013 (−0.130,0.157)	0.858	−0.117 (−0.731,0.497)	0.709
lnMn	−0.019 (−0.046,0.008)	0.166	−0.015 (−0.046,0.016)	0.345	−0.133 (−0.305,0.040)	0.132	−0.004 (−0.043,0.034)	0.826	−0.147 (−0.312,0.017)	0.079
lnFe	0.169 (0.064,0.274)	0.002	0.016 (−0.104,0.136)	0.792	−0.001 (−0.673,0.671)	0.998	0.153 (0.004,0.303)	0.045	0.015 (−0.625,0.656)	0.963
lnCu	0.068 (0.000,0.136)	0.049	0.164 (0.087,0.241)	0.000	−0.440 (−0.874,−0.007)	0.047	−0.096 (−0.192,0.001)	0.052	−0.276 (−0.690,0.137)	0.190
lnZn	−0.065 (−0.122,−0.007)	0.028	−0.058 (−0.124,0.007)	0.082	0.394 (0.026,0.762)	0.036	−0.007 (−0.089,0.075)	0.873	0.336 (−0.015,0.687)	0.060

Data are expressed as coefficients (β) and 95% CI.

Adjusted for age, gender, BMI, smoking status, triglycerides, total cholesterol, hypertension, diabetes, and urinary iodine.

Continuous variables with skewed distributions (Ca, Mg, Mn, Fe, Cu, Zn, FT3, FT4, TSH, FT3:FT4 ratio, and TT4RI) were ln transformed for analysis.

### 3.3 The association between selected metals and thyroid function biomarkers using the quantile g-computation model

There was no significant association between the metal mixtures and thyroid function biomarkers in the QGC models, and the individual weights for each metal were present (**Table 3**). Fe and Ca were identified as influential metals of FT3; Fe (weight = 0.576) had a positive contribution, and Ca (weight = 0.505) had negative weights. Regarding FT4, Cu (weight = 0.987) was a positive contributor, and Zn (weight = 0.553) was a negative contributor. Moreover, Zn (positive weight= 0.881), Cu (negative weight = 0.463), and Mn (negative weight = 0.448) contributed to TSH. It was found that Fe (weight = 0.671) had the greatest and most positive contributions to the FT3:FT4 ratio, and Ca (weight = 0.559) made more contributions than Cu (weight = 0.441) in the negative weights. Regarding TT4RI, Zn (weight = 0.864) was the most influential metal in the positive weights, and Mn (weight = 0.562) had a negative contribution.

**Table 3:** Weights of each metal and the metal mixture for thyroid function biomarkers using the QGC analysis.

Metals	lnFT3		lnFT4		lnTSH		lnFT3:FT4 ratio		lnTT4RI	
	QGC		QGC		QGC		QGC		QGC	
lnCa	0.505	Neg*	0.002	Neg	0.119	Pos	0.559	Neg*	0.136	Pos
lnMg	0.122	Pos	0.142	Neg	0.073	Neg	0.240	Pos	0.109	Neg
lnMn	0.203	Neg	0.303	Neg	0.448	Neg*	0.004	Pos	0.562	Neg*
lnFe	0.576	Pos*	0.013	Pos	0.016	Neg	0.671	Pos*	0.016	Neg
lnCu	0.219	Pos	0.987	Pos*	0.463	Neg*	0.441	Neg*	0.313	Neg
lnZn	0.291	Neg	0.553	Neg*	0.881	Pos*	0.086	Pos	0.864	Pos*
Element mixture *β* (95%CI)	0.003 (−0.013,0.018)		0.001 (−0.016,0.019)		−0.016 (−0.114,0.081)		0.002 (−0.020,0.023)		−0.015 (−0.108,0.078)	

Adjusted for age, gender, BMI, smoking status, triglycerides, total cholesterol, hypertension, diabetes, and urinary iodine.

Neg: weight in negative direction from QGC. Pos: weight in positive direction from QGC.

*Influential metals defined by QGC with absolute weight values >0.333.

Continuous variables with skewed distributions (Ca, Mg, Mn, Fe, Cu, Zn, FT3, FT4, TSH, FT3:FT4 ratio, and TT4RI) were ln transformed for analysis.

### 3.4 The association between selected metals and thyroid function biomarkers using the Bayesian kernel machine regression model

**Figure 3:**
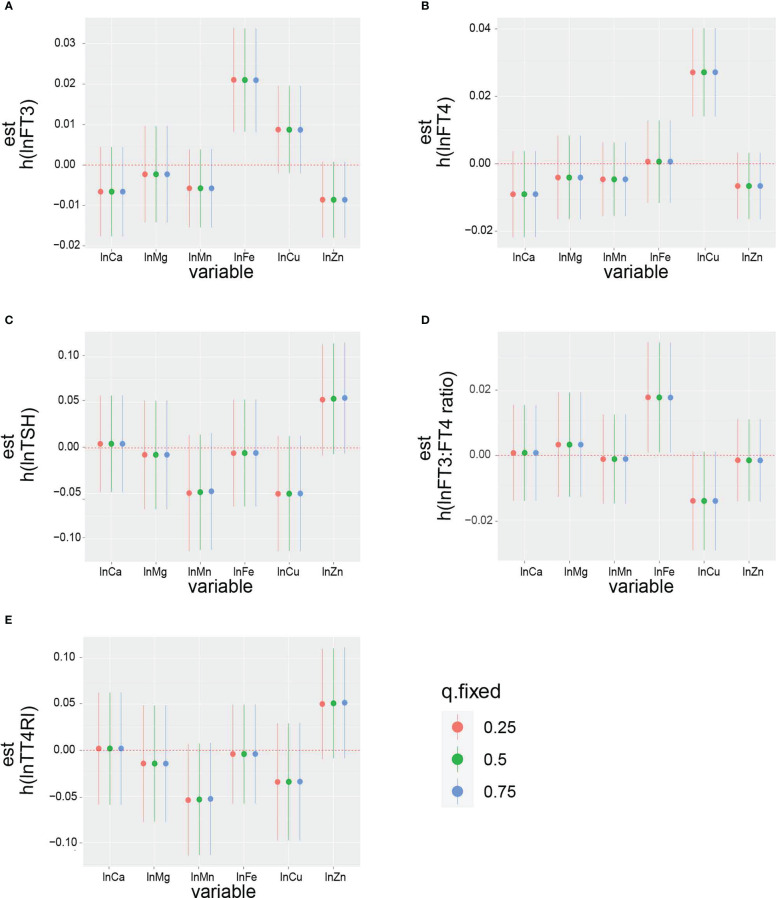
The impact of single exposure on: **(A)** lnFT3, **(B)** lnFT4, **(C)** lnTSH, **(D)** lnFT3:FT4 ratio, and **(E)** lnTT4RI when an individual metal was at its 75th percentile compared with when that exposure was at its 25th percentile, while all of the other metals were fixed at the 25th, 50th, or 75th percentiles. The results were adjusted for age, gender, BMI, smoking status, triglycerides, total cholesterol, hypertension, diabetes, and urinary iodine.

**Figure 4:**
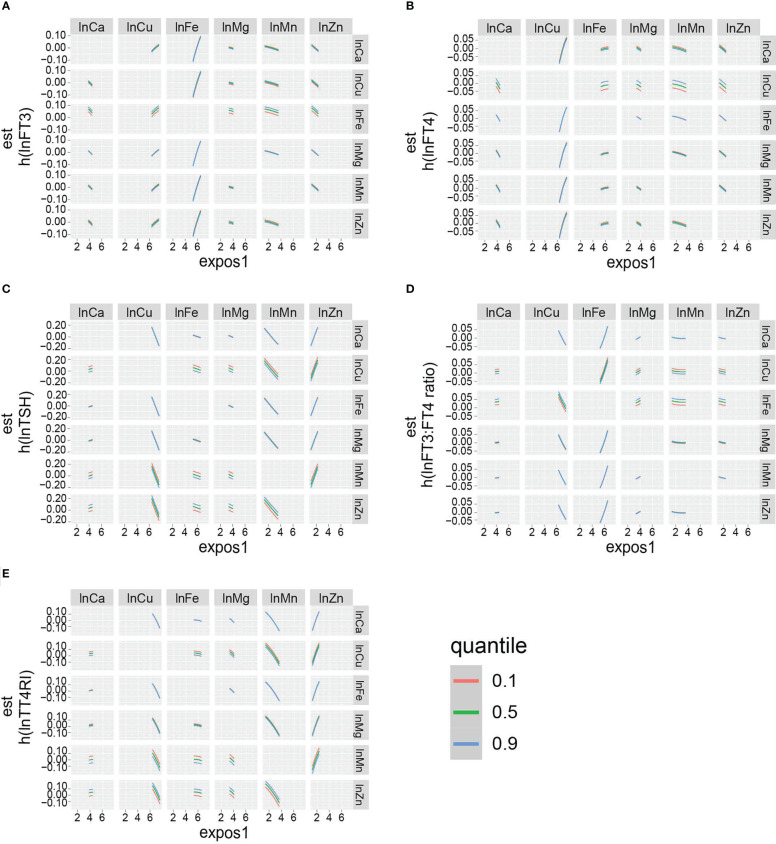
Bivariate exposure dose–response curves: dose–response curves for **(A)** lnFT3, **(B)** lnFT4, **(C)** lnTSH, **(D)** lnFT3:FT4 ratio, and **(E)** lnTT4RI of a metal when another metal is fixed at the levels of 10th, 50th, and 90th percentile. The remaining five metals are all fixed at the median. The results were adjusted for age, gender, BMI, smoking status, triglycerides, total cholesterol, hypertension, diabetes, and urinary iodine.

The overall effect of the selected metals on the thyroid function biomarkers was analyzed using BKMR (**Figure S1**). The metal mixtures were not statistically significant for thyroid function biomarkers. The rank of PIP in relation to the thyroid function biomarkers obtained from the BKMR model is summarized in **Table 4**. The highest PIP of FT3, FT4, TSH, FT3:FT4 ratio, and TT4RI was from Fe, Cu, Zn, Fe, and Zn, respectively. Additionally, the single exposure–response relation of each selected metal was similar to the findings from the linear regression (**Figure 3**). Moreover, there was no significant interaction between the studied metals on thyroid function biomarkers (**Figure 4**).

**Table 4:** Posterior inclusion probabilities (PIPs) estimated by BKMR.

Metals	lnFT3		lnFT4		lnTSH		lnFT3:FT4 ratio		lnTT4RI	
	PIP	rank	PIP	rank	PIP	rank	PIP	rank	PIP	rank
lnCa	0.266	2	0.252	2	0.482	6	0.326	4	0.406	4
lnMg	0.218	3	0.162	4	0.514	4	0.378	3	0.388	5
lnMn	0.064	6	0.062	6	0.582	3	0.144	6	0.516	2
lnFe	0.963	1	0.118	3	0.484	5	0.852	1	0.264	6
lnCu	0.167	4	0.906	1	0.668	2	0.612	2	0.421	3
lnZn	0.137	5	0.078	5	0.671	1	0.229	5	0.582	1

Adjusted for age, gender, BMI, smoking status, triglycerides, total cholesterol, hypertension, diabetes, and urinary iodine.

Continuous variables with skewed distributions (Ca, Mg, Mn, Fe, Cu, Zn, FT3, FT4, TSH, FT3:FT4 ratio, and TT4RI) were ln transformed for analysis.

## 4 Discussion

In this study, we used the linear regression, QGC, and BKMR methods to investigate the relative contribution of each metal in the combined exposure model. Alongside the results from the methods mentioned above, we propose that in an iodine-adequate area, TSH levels of general adults are correlated with blood Cu (negative) and Zn (positive). Blood Fe and Cu have the strongest correlation with FT3 and FT4 levels, respectively. Blood Zn was also negatively related to FT4. For thyroid hormone sensitivity indexes, the blood Fe concentration is positively related and blood Cu concentration is negatively related to the FT3:FT4 ratio in all methods and models. Moreover, we found that blood Mn was inversely correlated with TT4RI, while Zn had a positive relationship with TT4RI. Lastly, no significant association was found between the metal mixtures and thyroid function in the QGC and BKMR models.

The results calculated by the three combined exposure model methods are not completely consistent. For example, blood Ca was negatively related to serum FT3 and the FT3:FT4 ratio in QGC, but not linear regression or BKMR. Blood Zn had a negative relationship with FT3 in linear regression, but not QCG or BKMR. Currently, there is no consensus on which of the statistical methods is the best for mixture analysis. The purpose of QGC is to specifically address the inherent complexities of high-dimensional mixture data and to estimate the joint effect of the analyzed chemicals (24). BKMR can be used to find the nonlinear exposure–response relationship with other metals fixed at certain levels. However, it cannot evaluate the co-exposure patterns with both high and low levels of metals (23). Thus, current recommendations insist on conducting combined analyses using different approaches together.

Mn is an enzymatic cofactor and is found in a variety of enzymes, including oxidoreductases, transferases, hydrolases, lyases, isomerases, and ligases. Therefore, Mn plays an important role in energy metabolism, immune function, blood coagulation, hemostasis, antioxidant defenses, and other physiologic processes (25). It is an abundant metal that exists in the air, soil, and waterways, which is ultimately consumed by humans (26). Therefore, Mn deficiency is rare in normal conditions. On the contrary, Mn exposure induces neurocognitive deficits in fetuses and manganism syndrome in adults (27). In addition to neurotoxicity, Mn exposure impairs thyroid function. Investigations into how Mn affects neural endocrine hormones among welders and smelters have been conducted in China. The results showed that smelters have higher Mn exposure and lower TSH, prolactin, testosterone, and follicle-stimulating hormone serum concentrations (28). Moreover, Buthieau et al. treated rats with MnSO_4_ for 5 weeks and found a high accumulation of manganese in the pituitary gland, along with decreasing serum T4, T3, and TSH levels (29). In our findings, we observed no association between blood Mn and FT3 or FT4, but we found an inverse correlation between Mn and TT4RI, which indicates that less TSH had been released than expected according to the actual FT4 level. As our research was performed on the general population with normal blood Mn levels, it is presumed that a low dose of Mn exposure can reduce TSH release in the pituitary.

It has been well documented that Zn is an essential metal and is indispensable for human health. Zn deficiency has a detrimental impact on growth, neuronal development, and immunity. However, excessive Zn exposure through the skin, inhalation, or ingestion also impairs multiple systems in the human body, such as the nervous system, cardiovascular system, and respiratory system (30). Studies have shown that insufficient or excessive Zn levels result in thyroid disorders. A case–control study found that plasma Zn levels were lower in hypothyroid subjects and normal in the hyperthyroid group compared with the control group (31). In a study on pregnant women, women with hypothyroxinemia (FT4 below the reference range with normal TSH) had significantly lower plasma Zn concentrations than euthyroid women, and the plasma Zn level was positively related to FT4 (32). In animal research, rats that were given Zn-deficient diets had a lower serum T3 level than the control group and showed signs of apoptosis and severely altered follicle cellular architecture in thyroid tissue (33). In contrast, Jain et al. showed that the level of Zn is associated with decreased levels of FT4 and TT4 in men (19). Similar results were found in our study, as we discovered that blood Zn was positively related to the TSH level and inversely correlated with the concentration of FT4. Zinc chloride was found to be a thyroid hormone receptor antagonist, as it can hinder the binding of the thyroid hormone(34), which may explain the positive relationship between blood Zn and TT4RI in our findings. Moreover, several studies found that the Cu level is correlated with increased FT4, which is the same result as in our study (19, 35). Moreover, we found that blood Cu is correlated with decreased TSH and the FT3:FT4 ratio. An interaction exists between thyroid hormones and Cu; an animal experiment proved that the thyroid hormone regulated the serum Cu level by promoting the synthesis and export of hepatic copper-transport protein (36). Cross-sectional research on children who had been diagnosed with congenital hypothyroidism showed that the serum level of Cu was strongly associated with thyroid hormones, and severely hypothyroid children have a higher risk of Cu deficiency (37). On the other hand, Iseki et al. found that intracellular redox-active Cu regulates the proliferation and expression of Pax-8 and thyroid peroxidase (TPO) in thyroid follicular cells (38). Furthermore, rats that were fed a Cu-deficient diet had an impaired response to serum T4 but not to TSH or TRH compared with the control group, which indicated that Cu is crucial for T4 synthesis or release (39). Thus, we surmised that Cu levels affected TSH levels and FT3:FT4 ratio by increasing FT4 levels. Fe is the most abundant transition metal in the human body, and Fe deficiency usually results in anemia. Results of a cross-sectional study indicated that hypothyroidism was common in Fe-deficient and anemic children. Supplementing Fe and iodized salt simultaneously improves the efficacy of iodine in Fe-deficient goitrous children (15, 16). In goitrous female patients, a positive correlation between serum Fe and TSH, FT3, and FT4 levels was observed by Kandhro et al. (40). Biologically, TPO, which is essential for thyroid hormone production, is a heme-containing enzyme, and its activity is dependent on Fe. Interestingly, active T3 has been found to modulate hepatic ferritin expression, which is important for Fe absorption (11, 12). In this study, we found that blood Fe is positively associated with FT3 but not FT4. Moreover, the results showed that Fe has the greatest positive correlation with the FT3:FT4 ratio. Previous studies found that reduced rates of T3 production might be due to decreased liver deiodinase activity in Fe-deficient rats (41).

The advantage of our study is that we adopted various methods, including traditional (linear regression) and advanced statistical methods (BKMR and QGC), to assess the impacts of selected metals on thyroid function in combined model, which could suggest the potential interactions among metals and is still sparse. However, our study also had several limitations. One limitation is that this is a single-center study and all subjects are from Guangdong, but this does not affect the credibility of the conclusions as our sample is sampled strictly according to population stratification. Another limitation of our study is the shortcomings of the cross-sectional study, as we could show the correlation between the selected metals and thyroid hormone but not the causality. Moreover, we could not analyze the metal exposure in detail, such as the duration and sources, which may have different effects.

## 5 Conclusions

We have provided evidence for associations between selected metals and thyroid function. As shown above, we suggest that Mn, Fe, Cu, and Zn levels were correlated with thyroid function.

Patients with thyroid disorders are recommended to measure Mn, Fe, Cu, and Zn levels and determine whether they also have excess or deficiency of those metals. Further studies are needed to understand the relationship between metals and thyroid function.

## Data availability statement

The original contributions presented in the study are included in the article/**Supplementary Material**. Further inquiries can be directed to the corresponding authors.

## Ethics statement

The studies involving human participants were reviewed and approved by Ethics Committee of Shunde Hospital, Southern Medical University, Shunde, Foshan, China (20211103). The patients/participants provided their written informed consent to participate in this study.

## Author contributions

Conceptualization: JS and HW. Methodology and formal analysis: YeL and QM. Resources: JS. Data curation: YeL. Writing—original draft preparation: YY. Writing—review and editing: HW. Supervision: JS and HW. Project administration: JS. Funding acquisition: JS. Investigation and data acquisition: All authors. All authors contributed to the article and approved the submitted version.

## Supplementary material

Supplementary Figure 1 (Figure S1) Overall effect (95% CI) of selected elements on: **(A)** lnFT3, **(B)** lnFT4, **(C)** lnTSH, **(D)** lnFT3:FT4 ratio, **(E)** lnTT4RI when all the elements at particular percentiles were compared with all the metals at their 50th percentile. The results were adjusted for age, gender, BMI, smoking status, triglycerides, total cholesterol, hypertension, diabetes, and urinary iodine.
