# Supplementary figures and images for: Association of multiple blood metals with thyroid function in general adults: A cross−sectional study
Source: Front Endocrinol (Lausanne). 2023 Mar 27;14:1134208. doi: 10.3389/fendo.2023.1134208 (PMC10083359; doi:10.3389/fendo.2023.1134208)

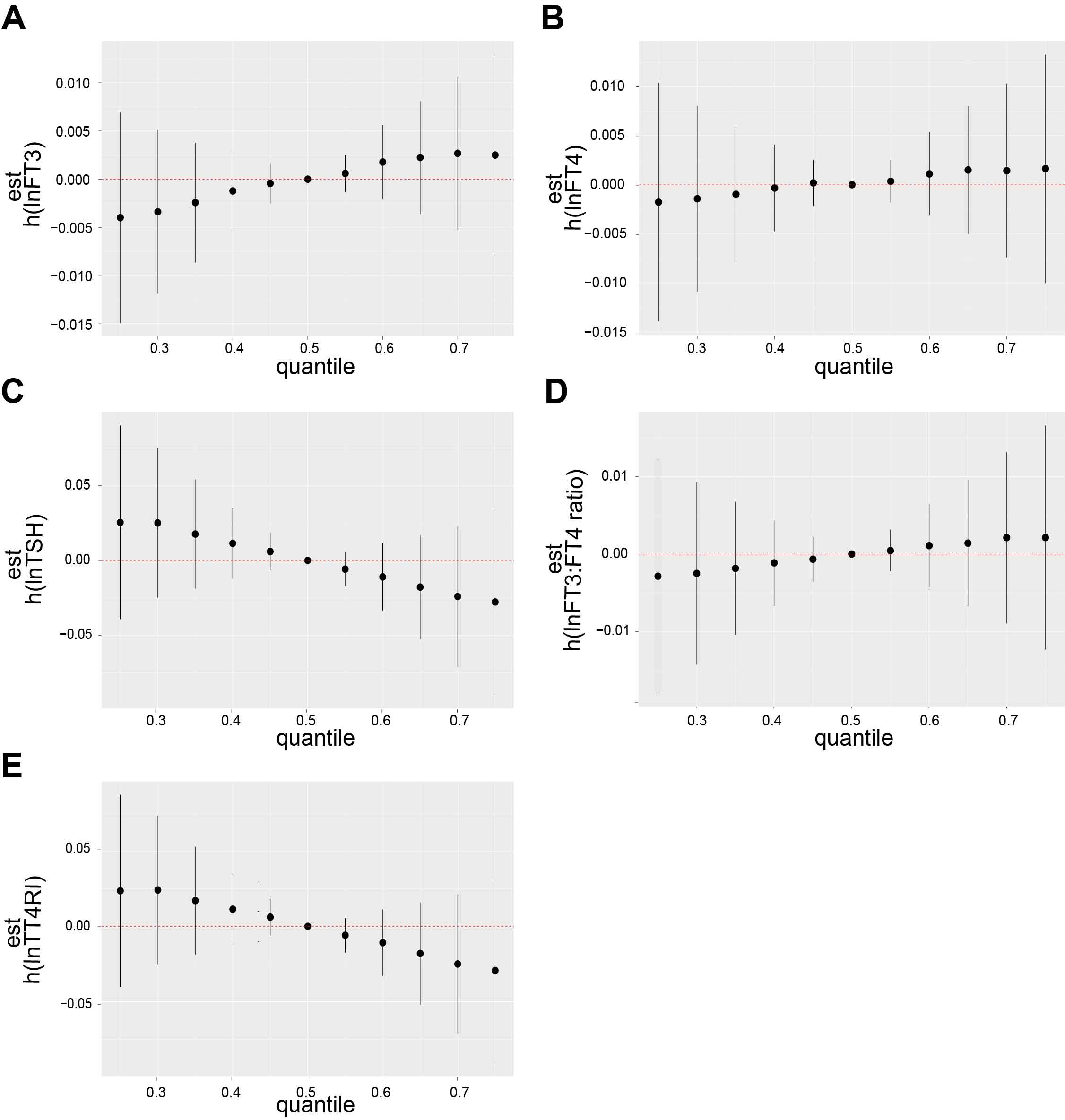

Supplement: Supplementary Figure 1 — (Figure S1) Overall effect (95% CI) of selected elements on: (A) lnFT3, (B) lnFT4, (C) lnTSH, (D) lnFT3:FT4 ratio, (E) lnTT4RI when all the elements at particular percentiles were compared with all the metals at their 50th percentile. The results were adjusted for age, gender, BMI, smoking status, triglycerides, total cholesterol, hypertension, diabetes, and urinary iodine. [file Image_1.jpeg]
